# Exploring the Intersection of Lakescapes and Military History

**DOI:** 10.1177/25148486251363734

**Published:** 2025-08-03

**Authors:** Elodie Charrière

**Affiliations:** Department of Social Sciences, Michigan Technology University, USA; Environmental Governance and Territorial Development Hub, Institute for Environmental Sciences, 27212 University of Geneva, Switzerland

**Keywords:** Lakescape, militarization, legacy, history

## Abstract

The study of processes leading to the militarization of landscapes is a rapidly expanding field of research. While certain themes, such as the impact of conflicts on landscape transformation, have received significant attention, others remain comparatively under-examined. Specifically, the environmental consequences of military training activities on inland freshwater ecosystems in the context of war preparedness have been largely overlooked by historians. This topic, however, falls within the dimension of militarized landscapes, encompassing lakescapes and the related environmental processes that undergo transformation due to conflict preparation. This article aims to address this gap by exploring a specific typology of underwater munitions sites: former military training targets and ranges located within inland freshwater bodies, with a particular focus on Lake Michigan in the United States and Lake Neuchâtel in Switzerland. By contextualizing these sites within a historical framework and examining their commonalities and differences in training practices, this interdisciplinary research will discuss the current management—or lack thereof—of this military legacy resting at the bottom of the lakes. The aim of this research is to highlight how lakescapes have been transformed by military training and how such practices have environmentally impacted inland freshwater bodies. This study finds that these military activities were not concealed from the public, that they have environmental consequences, and that they consistently receive less attention than terrestrial sites.

## Introduction

The concept of militarized landscapes encompasses a range of situations that share a common characteristic: they are partially or fully mobilized to achieve military objectives. In the afterword of the book “Militarized Landscapes” (Pearson et al., 2010), Russell (2010) suggests that researchers interested in this field should broaden their understanding of military landscapes beyond the traditional images of battlefields and military bases. This special issue aligns with this suggestion by expanding the scope of militarized landscapes to include “landscapes and related environmental processes that are transformed by political hostilities, preparation for conflict, and outright war, as well as landscapes militarized to ostensibly protect natural resources or otherwise slow or reverse environmental change” (Lunstrum and Brady, 2025 In review). In doing so, this article explores the interrelations between military training activities and lakeshores, as well as lake ecosystems, from the period of twentieth century war preparedness to the present day.

Investigating the war preparedness period is relevant for two main reasons. Firstly, as noted by Machlis and Hanson (2008) and later emphasized by McNeill (2021), the environmental consequences of war preparations often surpass those of actual combat. Secondly, as contributions to this special issue demonstrate, no part of the world has a monopoly on environmentally impactful military activity. War preparedness is not limited to major belligerent powers—nations capable of initiating or engaging in war, such as the United States during the Second World War, the Vietnam War or the Cold War—but also extends to neutral countries like Switzerland. War preparedness can encompass vastly different situations, preceding the actual war and occurring within a strategic framework of rearmament, or it can be constructed during the implementation of the conflict. Lastly, war preparedness does not necessarily mean that war is inevitable.

First introduced by Woodward (2004) and later revisited by Pearson (2012), scholars from various disciplines have only recently begun to extensively explore the relationships between war, landscape, militarization, and the environment. Since then, research in this area has continued to expand. Many of these studies have focused on the interactions between training bases and shooting ranges and their surrounding environments, particularly in the United States (Doe III, 2011a, 2011b; Edgington, 2014; Gibbes et al., 2017; Martini, 2015), as well as in other regions of the world (Biggs, 2018; Dudley, 2013; Sanderson et al., 2017; Šantrůčková et al., 2020; Zentelis et al., 2017). However, there are limited studies dedicated to examining the environmental disturbances caused by military training in aquatic environments. In the context of military activities, the term “aquatic space” encompasses two categories: tidal water and inland water. Scholars have studied ranges and targets areas in oceanic environments, such as Vieques Island (Davis et al., 2007; Dycus, 1996; Grusky, 1991; Márquez and Fernández Porto, 2000), but they have given less attention to those located in inland freshwater bodies.

This omission raises an important question: why have lake and other inland waters been largely excluded from the analysis of militarized environments? One possible explanation lies in the enduring conceptual divide between military and civilian spaces. Yet scholars have increasingly challenged this dichotomy. Focusing on the intricate entanglements between the military and the environment, Davis (2007) asserts that “militarized landscapes extend far beyond combat zones and even the carefully delineated properties of military facilities” (133). Similarly, Reno explores how American citizens engage with military waste outside the official structures of the U.S. defense establishment. He contends that “just as war preparation impacts people outside of formal war zones, it also comes into the lives of people outside of formally militarized spaces like testing grounds, factories, laboratories, and bases” (Reno, 2019: 1).

Adopting this broader spatial lens highlights the importance of including all forms of natural environments—terrestrial and aquatic, saline and freshwater—within the scope of militarization studies. Omitting inland freshwater bodies renders this perspective incomplete and risks erasing both the contemporary and historical impacts of military activity on aquatic ecosystems. This approach echoes Havlick's (2018) call to engage with former military sites in their full historical, social, and ecological complexity to prevent their depoliticization and loss of meaning. Furthermore, Cumming (2024) notes that underwater munitions—whether resulting from warfare, dumping, surplus disposal, or underwater training ranges—pose a contemporary challenge, as certain sites have been identified as sources of contamination and/or risk. Thus, studying underwater ranges and target areas within inland freshwater bodies is essential for informed risk assessments, long-term monitoring, and public awareness.

To better understand the interconnections between military activity and inland freshwater, this study draws on the concept of waterscape, a term that has evolved significantly over time. Originally used to describe works of art depicting water-based scenery (Orlove and Caton, 2010), the term gained scholarly prominence in the late twentieth century, particularly through Swyngedouw's (1999) definition of waterscapes as hybrid spaces—simultaneously natural and social—shaped by complex historical-geographical processes. Since then, scholars across a range of disciplines—including political ecology (Flaminio et al., 2022), hydrology (O'Sullivan et al., 2021), water history (Hundley, 1987), environmental psychology (Herzog, 1985), and urban planning (Dreiseitl and Grau, 2009)—have embraced the concept. This interdisciplinary engagement has led to diverse interpretations (Bouleau, 2014; Hurst et al., 2022; Karpouzoglou and Vij, 2017), but they share a common aim: to improve our understanding of the water-space-society nexus.

As the concept has evolved, scholars have developed more specific terms— riverscape (Stanford et al., 2017), pondscape (Cornea et al., 2016), and lakescape (Bardati, 1996)—to examine how different water bodies both shape and are shaped by human activities. The term lakescape, for example, has been used to investigate socio-environmental practices such as tourism (Potocka, 2013) and archaeology (Minler et al., 2011). This article contributes to that growing literature by applying the concept of lakescape to the underexplored context of military training, revealing a unique convergence of environmental change and militarization.

The article's originality lies in its focus on the nexus between military activity and environmental transformation, interpreted through the concept of lakescape. In this study, lakescape is understood as a dynamic and hybrid socio-natural formation shaped by past and present military activities. The term lakescape refers not only to the physical body of water and its ecosystem but also to the surrounding shoreline and lakebed, where traces of military training may persist. These sites are more than ecological zones; they are also historical, cultural, and political spaces where the legacy of war preparedness is materially embedded. By highlighting these footprints—some visible on land, others concealed underwater—this article aims to offer a fuller account of the long-term environmental consequences of military infrastructure and activity in inland freshwater environments.

To examine these dynamics, the article analyses two military training activities—shooting ranges and target areas—through two case studies: Lake Michigan in the United States and Lake Neuchâtel in Switzerland. Both lakes have historically served as military firing ranges, with the latter still in use today. Despite differing military policies, studying the training activities conducted in these lakes—including their use as targets for the air force and as shooting ranges—provides insight into the evolution of the relationship between military activities and lakes from the twentieth century to the present day. The military legacy of both Lake Neuchâtel and Lake Michigan is extensive, encompassing military wrecks, such as aircraft (Gächter et al., 2004; Neyland, 2002), and a German submarine in the case of Lake Michigan (Wise, 1989).

This article aims to shed light on how military training reshaped lakescapes, as well as the environmental consequences of such practices on inland freshwater bodies. To accomplish this, this paper is structured into three sections. The first section provides context regarding military training activities conducted along the shores of Lake Michigan, including lake shooting areas and former bombing and target areas over water. The second section focuses on military activities carried out on Lake Neuchâtel, such as the Forel aviation firing range and artillery test firings. The final section examines the current state of underwater munitions management at both sites. Despite a common solution applied to these two case studies—allowing the munitions waste to rest in the lake's bottoms—the process leading to this outcome varies depending on the specific characteristics of each training site. Therefore, this analysis highlights the importance of acknowledging and addressing the environmental legacies of militarized lakescapes to better protect global freshwater ecosystems.

## Lake Michigan

The Great Lakes region of North America, encompassing both the American and Canadian sides, bears the legacy of past or current military activities, including munitions plants, training sites, depots, and bases (e.g., Jenks, 2007; MacFarlane, 2024: 141–142; Thomson and Mayo, 1991). This article specifically focuses on Lake Michigan, the third-largest of the Great Lakes, with a surface area of 22,300 square miles, an average depth of 279 feet, and a maximum depth of 923 feet (Ashworth, 1987). Lake Michigan lies entirely within the United States, bordering the states of Michigan, Wisconsin, Illinois, and Indiana. The volume of the lake is 1180 cubic miles, and the water retention time is 62 years.

As part of the national program known as “Formerly Used Defense Sites” (FUDS), the U.S. Army Corps of Engineers (USACE), acting on behalf of the U.S. Army and the Department of Defense (DoD), catalogs all properties owned, leased, or otherwise held by the United States under the purview of the Secretary of Defense prior to October 17, 1986 (Regulation No. 200–3–1). Its aim is to carry out environmental restoration efforts on these properties. Through this program, the USACE has documented the extensive history of military activities throughout the United States^1^, identifying eleven underwater munitions sites in Lake Michigan. The concept of underwater munitions sites, developed by the DoD, encompasses seven categories^2^, all of which pose threats to human safety and the environment. In the context of Lake Michigan, the category “ranges and target areas” applies.

**Figure 1.**
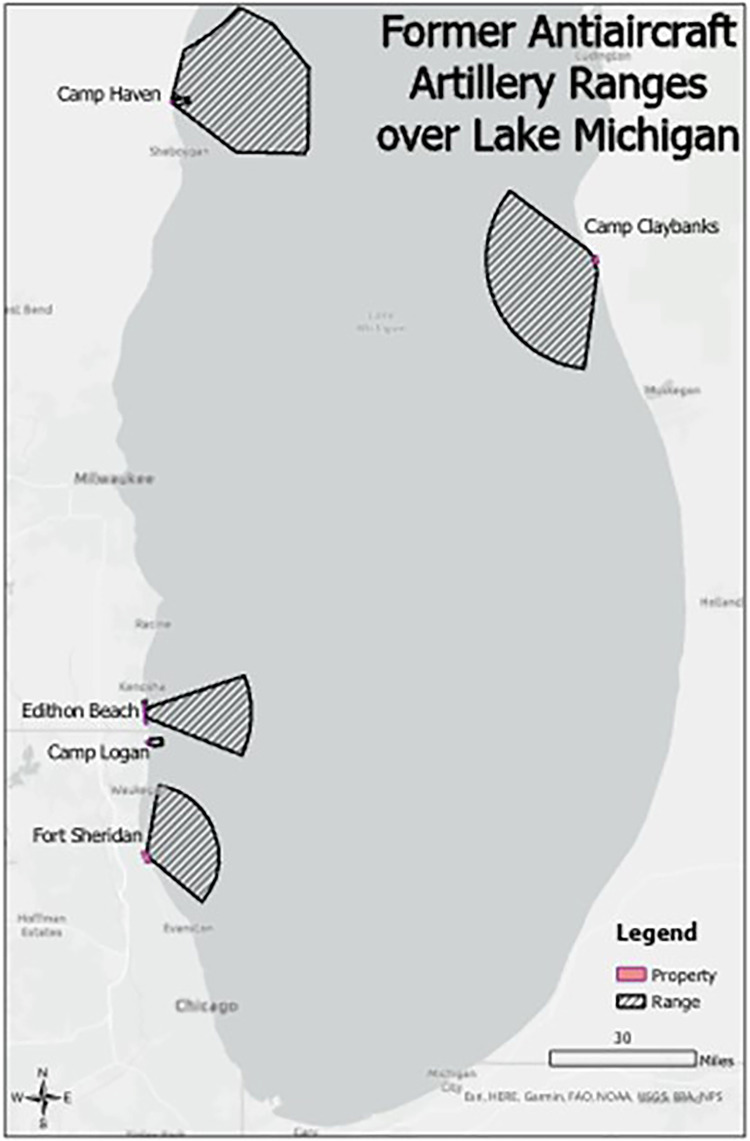
Former Anti-aircraft Artillery Ranges over Lake Michigan.

Overall, U.S. military ranges listed within the FUDS program are primarily concentrated in tidal water areas, but eight of them are located on the shores of the Great Lakes in inland water areas (SERDP and ESTCP, 2010). Specifically, Lake Michigan is most affected, with five former anti-aircraft artillery (AAA) ranges located on its shores (Figure 1): Camp Claybanks (Michigan), Camp Haven (Wisconsin), Camp Logan (Illinois), Edithton Beach (Wisconsin), and Fort Sheridan (Illinois). Two of them—Fort Sheridan and Camp Logan—were established in the late nineteenth century, in 1887 and 1892 respectively, and were used for various military purposes (Dretske, 2007; USACE, 1996). In contrast, the other three ranges—Camp Claybanks, Camp Haven, and Edithton Beach—were established between World War II and the early 1950s and were exclusively used for AAA training (Sicard, 2018; USACE, 2002a, 2002b). Indeed, anti-aircraft guns were a prominent weapon during WWII and were employed both on land and on sea as a defense against aerial attacks, as well as against enemy submarines and tanks.

To simulate wartime conditions, AAA training sessions on Lake Michigan were conducted both during the day and at night, with nocturnal exercises assisted by artillery searchlights. Trainees fired at conical cloth targets—each approximately 40 feet in length—towed by aircraft flying at altitudes of up to 20,000 feet and speeds of 175 mph, as well as at both fixed and mobile targets positioned over the lake (Chicago Daily Tribune, 1940a, 1940b). A broad array of anti-aircraft weaponry was employed, including .30- and .50-caliber machine guns, 20 mm to 120 mm artillery, and 3-inch cartridges. These weapons were operated from batteries strategically stationed along the shoreline and oriented toward the lake to engage airborne targets effectively.

Although training methods and equipment varied, all AAA training sites shared a key feature: they conducted firing exercises over Lake Michigan, resulting in the creation of water ranges. As a result of the extensive use of ordnance—including 37 mm shells, 120 mm projectiles, and 3-inch cartridges—these ranges, which varied in depth and surface area, now contain munitions debris and unexploded ordnance (UXO). Despite differences in location, scale, operational periods, and users, this environmental legacy is consistent across sites. AAA training practices began in the early twentieth century, peaking in the 1940s and early 1950s, but rapidly declined in the late 1950s, with the last training exercises taking place at Camp Haven in 1959 (Dippel, 2017). This rendered AAA units obsolete and replaced them with units handling guided missiles.

All of these military sites are now closed. Edithton Beach was the first to close in 1946 after World War II (USACE, 2002a), and the most recent closure occurred in 1993 when the Defense Secretary's Commission (1988: 59–60) designated Fort Sheridan for closure under the Base Realignment and Closure Act. After closure, these former military sites underwent various developments. Camp Claybanks’ land was transferred for residential and recreational purposes (USACE, 2002a), while Camp Logan's land became part of the Illinois Beach State Park under the Illinois Department of Conservation (GEO Consultants, 2009). Edithton Beach's land was divided and sold to developers for residential housing and was converted into a public beach area by the Wisconsin Department of Natural Resources (Shaw Environmental, 2009). Camp Haven, after being acquired by Wisconsin Electric, was resold to Kohler Inc., and transformed into the Whistling Straits golf course (USACE, 2002b). Fort Sheridan was divided into three distinct areas, with portions transferred to the U.S. Reserve Army Command, the U.S. Navy, and the municipalities of Highland Park and Highwood for urban development and to the Lake County Forest Preserve District for recreational activities (Engineering-environmental Management, 2005).

In addition to these AAA training sites, five islands located in the northeastern part of Lake Michigan, served as targets and bombing areas during World War II: Hat Island, Hog Island, Pismire Island, Shoe Island (also known as Shore Island), and Waugoshance Point (a property on Waugoshance Island, formerly known as Crane Island). On June 22, 1944, the Department of Interior withdrew a parcel of land in Michigan, totaling 2513.54 acres, for the Navy's use as target areas for aerial bombing training (Public land order 237). The U.S. Naval Air Station at Traverse City, Michigan, used these five islands as aviation targets for high-altitude bomb tests. In October 1945, the Navy discontinued training activities at the Naval Air Station at Traverse City, reassigning operations to Mojave, California (Shaw Environmental, 2011: Appendix B: 323). The Headquarters, Commander Naval Air Bases and Ninth Naval District terminated the Navy's lease on these islands in June 1946 (HydroGeoLogic, 2013: Appendix G.06). It was not until April 2, 1956, that the Department of Interior officially revoked the public land order 237 (Public land order 1279). Since then, all of these islands have been returned to public lands and are now part of natural reserves. Specifically, the U.S. Fish and Wildlife Service, Region 3, Michigan Islands National Wildlife Refuge manages Shoe Island, Pismire Island, and Hat Island. Hog Island is part of the Mackinaw State Forest, while Waugoshance Point is located within the Wilderness State Park.

## Lake Neuchâtel

To safeguard its independence and security, the principle of neutrality has been an integral component of Switzerland's foreign policy since its establishment in 1848 (Federal Constitution of 1848). However, the adoption of the Federal Constitution of 1874 transferred responsibility for foreign policy and national defense, including oversight of military training, to the federal government. Despite the country's commitment to neutrality, the Swiss military must remain prepared to safeguard the nation's territory and uphold the integrity of its borders. Consequently, despite its neutrality and the absence of combat on its territory during the two World Wars, visible military remnants exist on Swiss soil. Historical studies have documented strategic constructions from the first half of the twentieth century, such as forts (Pike, 2017) and “Toblerones”, a type of anti-tank barrier (Ross, 2012).

This article focuses on the relationship between military activities and aquatic resources, specifically addressing one of Switzerland's distinctive topographic features: the presence of over 1500 lakes within its territory. In 2004, responding to a political initiative (Haller, 2004), the Swiss Federal Department of Defense, Civil Protection, and Sport (DDPS) commissioned various research offices to compile a historical inventory of munitions and other military waste dumped into Swiss lakes. The objectives were twofold: (i) to document all deposits of munitions from the Confederation's army and armaments industry in the lakes, and (ii) to identify all target locations in the lakes used for military shooting exercises. The study concluded that approximately 8000 tons of munitions were dumped in four lakes by the military, and twenty-five lakes were used as shooting targets for the training of air forces, artillery units, and for testing by federal arms companies (DDPS, 2004).

Among these twenty-five lakes, Lake Neuchâtel received the largest quantity of training munitions due to firing exercises, primarily conducted by the air force and to a lesser extent by the military technical service's activities. Lake Neuchâtel is the largest entirely Swiss lake, with a surface area of 83 square miles, an average depth of 210 feet, and a maximum depth of 502 feet (Egloff and Kern, 2010). It borders the territories of four cantons: Neuchâtel (33.2 sq mi), Vaud (28.6 sq mi), Fribourg (20.5 sq mi), and Berne (0.8 sq mi). The lake's volume is 3.4 cubic miles, and its water retention time is 8 years.

**Figure 2.**
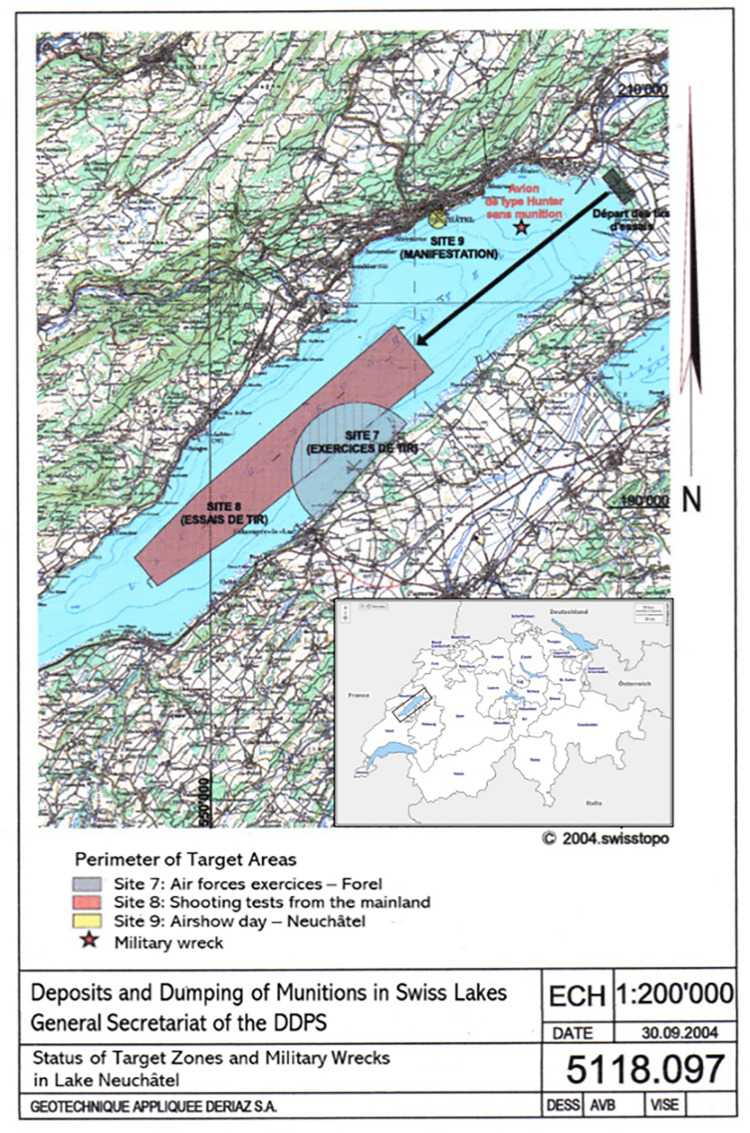
(Bottom right) Map of Switzerland with Lake Neuchâtel marked by a black rectangle. (Center) Underwater munition sites in Lake Neuchâtel. Adapted and translated from map 5118.097 (Gächter et al., 2004: Appendix A3).

Lake Neuchâtel contains three underwater munition sites (Figure 2).

The first site, the Forel firing range (Site 7 in Figure 2), covers an area of approximately 6.6 square miles and has experienced three distinct periods of activity. Initial aviation shooting training by the Swiss military began in 1924, with the first shooting sessions on Lake Neuchâtel occurring in 1928, following the establishment of the military airfield near Payerne in 1921. The first period extended from 1928 to 1950, saw the air forces utilizing between 50 and 500 tons of munitions. These primarily consisted of live-fire munitions, including machine guns, bombs, and rockets, along with a small number of inert munitions—munitions devoid of any explosive charge or priming. The second period began in 1950 and continues to the present day. During this phase, the Swiss air force utilized 4500 tons of inert munitions (Suter, 2004). Additionally, the air force employed 1 to 10 tons of live-fire munitions and 1.5 tons of guided missiles. The third phase spans from 1942 to 1999, corresponding to the use of 1 to 10 tons of 20 mm caliber projectiles by anti-aircraft defense units (Philipp, 2004). In total, approximately 5000 tons of munitions—primarily inert munitions and a smaller proportion of live ordnance—from military aviation and anti-aircraft defense exercises in Payerne are located within this area.

In addition to the large quantity of munitions received by Lake Neuchâtel, another characteristic sets it apart from other lakes used for military training: the length of operational periods. Thus, the army utilized Alpnachersee, a branch of Lake Lucerne, for an extended period (from 1950 to 2002), Lake Gruyère for a brief duration (1962 to 1965), and Lake Zurich (1979) and Lake Schiffenen (1994) on a single occasion each (Gächter et al., 2004: Appendix A4.5). Regarding Lake Neuchâtel, the practice at the Forel firing range began in the 1920s, peaked in the 1950s, and gradually declined, as evident in the decreasing number of firing days and rounds fired over the years. For example, in 1936, the air force dedicated 86 firing days at the Forel firing range, compared to 140 in 1937 (Gächter et al., 2004: Appendix A4.7). “In 1950, there were 197 firing days with 190,000 rounds fired, whereas in 1970, there were 137 firing days with 81,000 rounds fired, and in 1990, 78 days with 45,000 rounds fired” (Armée Suisse, 2021: 60). In the 2000s, the military aviation continued to use it for an average of twenty days per year, compared to the current usage of ten days per year (Forces aériennes, 2009). Despite recent political calls for its closure (Fridez, 2021) and remediation (Hurni, 2021), the Federal Councilor in charge of the DDPS justifies its continued use for the following reasons: (i) Forel is the only site where air force pilots can practice precision on moving targets; (ii) military aviation has long used only inert munitions; (iii) based on the current state of knowledge, there is no immediate risk to Lake Neuchâtel posed by munition residues (Amherd, 2022).

The second site (Site 8 in figure 2), used as a target area since 1950 by Armasuisse (formerly known as the Military Technical Service), covers approximately 21.2 square miles. Estimating the quantity of munitions in this area is challenging due to incomplete records regarding rounds fired and munition recovery operations following certain firing periods (Gächter et al., 2004). An approximate range of 1 to 100 tons can be assumed.

The third site (Site 9 in figure 2) resulted from a single day of military aviation exercise demonstrations conducted in 1986. During this event, the air force discharged 0.85 tons of inert munitions within an undisclosed perimeter (Suter, 2004).

## Military activities and lakescapes: What relationship?

The concise description provided illustrates that both Lake Michigan and Lake Neuchâtel bear the footprint of military training operations, not only on the shoreline but also underwater. This section aims to delve deeper into the complex and underexplored relationship between these activities and the lakescapes. The section is structured around three key themes that underscore the complexity and long-term effects of military-environmental interactions. The first theme emphasizes that, unlike many other instances, these training activities were not conducted covertly. The second theme focuses on the environmental and health risks associated with underwater munitions sites. Finally, the last theme highlights the distinctive characteristics of firing areas, former bombing sites, and target areas over water, which have become enduring legacies of military activities.

### Visible military training sessions

While Woodward (2010) points out that many military landscapes are shrouded in secrecy and hidden from civilian view, this is not the case with the shooting and target areas over Lake Michigan and Lake Neuchâtel. These areas present two distinct situations. A minority of them, specifically the islands used as bombing targets during World War II, can be considered concealed from civilians due to their isolation and distance from habitation. However, in the immediate post-war period, some islands retrospectively displayed their connection to past military activities. Two danger areas were marked on a navigation chart, one around Waugoshance Point, and the other including the east coast of Garden Island, Hog Island, and Hat Island (HydroGeoLogic, 2013: Appendix G.07). In addition, warning signs were deployed on Waugoshance Point to alert the public, particularly hunters and fishermen, to the presence of unexploded bombs (HydroGeoLogic, 2013: Appendix G.07). Similarly, warning signs were erected on Hat Island to prohibit public access due to the presence of UXO (Shaw Environmental, 2011: Appendix B). In contrast, most other locations, such as the training centers on the shores of Lake Michigan, as well as the air force's firing range and artillery testing area on Lake Neuchâtel, were situated in plain view and in close proximity to urban areas. Given this closeness, it is likely that local populations could hear the sounds of gunfire and military exercises. Furthermore, the Army occasionally organized public demonstrations of firing exercises over the lake, as seen at Fort Sheridan (Hutchings, 1940; Thomis, 1939) and Camp Haven (Sheboygan County Historical Research Center, 2019). Demonstrations also took place on Lake Neuchâtel for official visits, including that of Emperor Haile Selassie I of Ethiopia to Switzerland in 1954 (Gazette de Lausanne, 1954).

Following the approach of scholars who have demonstrated that news media play key roles in constructing public knowledge (Heinz, 2005; Lahtinen and Vuorisalo, 2004), the analysis of newspaper articles can be interpreted as evidence of civilian awareness of these military activities. In the late 1920s, the Gazette de Lausanne (1929) revealed Swiss aviation training on Lake Neuchâtel. In the 1930s and 1940s, numerous articles appeared in the local press describing AAA training conducted at Fort Sheridan (Chicago Daily Tribune, 1931, 1949) and Camp Logan (*The True Republican*, 1931). Similarly, in the early 1950s, The Montague Observer (1953) reported the establishment of a new AAA training center named Camp Claybanks. Articles accompanied by maps were also published to inform the local population about danger zones and areas restricted from navigation during exercise periods (see *The Sheboygan Press*, April 10, 1956, for Camp Haven, and the *Chicago Daily Tribune*, April 13, 1961, for Fort Sheridan). This practice continues in Switzerland today through two tools: (i) the display of posters specifying the dangerous areas of the lake, as well as the training schedules and days; (ii) the updating of a web page dedicated to aviation shooting notices by the Air Force.^3^

Thus, contrary to the perception shared by some scientists studying underwater munitions, who often assume that the dumping and/or training activities were carried out in secrecy under the guise of national security (Brewer and Nakayama, 2008) and without public knowledge, the case studies related to lake training demonstrate that these activities were well known, and the public was aware, though likely not of the specific details. This situation was also widely observed in the aftermath of World War II, a period when governments and militaries were eager to publicize dumping practices (Souchen, 2021: 362). At that time, numerous newspaper reports documented the disposal of surplus munitions while attempting to alleviate public apprehension and anxiety regarding these operations. For instance, reports highlighted the dumping of bullets into Lake Superior (Duluth News-Tribune, 1945), the disposal of obsolete ammunition in Georgian Bay, Lake Huron (Vipond, 1945), and the immersion of poison gas in the sea east of Ireland (New York Times, 1945).

However, despite the transparency associated with these operations, the relationship between the civilian and military communities was not always harmonious. After World War II, the population residing on the shores of Lake Michigan began to express dissatisfaction and opposition to these activities for various reasons, including the noise of the practice and the restrictions on navigation in certain areas of the lake during training (Chicago Daily Tribune, 1953; Thompson, 1955). The strongest opposition emerged in the early 1950s and centered around the construction of Camp Claybanks. Local and seasonal residents, who had chosen to enjoy the natural spaces of Lake Michigan, led the opposition to this site (Turner, 1953). This situation echoes the argument developed by Langston (2021), who suggests that a post-World War II economic boom, coupled with a newfound interest in the outdoors, led to significant lakeshore tourism and development in the Great Lakes region. However, the local population never mentioned the potential environmental consequences that such practices could have on the lake's fauna and flora. Similarly, after the closure of these sites, no concerns emerged regarding the presence of underwater UXO at the bottom of the lake. In Switzerland, the situation differs, as an area of Lake Neuchâtel is still utilized as a firing range. Therefore, conflicts of use between the DDPS, farmers, local residents, environmental protection associations, as well as fishermen, continue to persist (Forces aériennes, 2009). This situation is not unique, as in the United States, land management has often clashed with military training needs (Doe III, 2011b).

### Environmental consequences

Numerous studies have been conducted on military training grounds (military shooting ranges) to assess their environmental impacts—such as soil and groundwater contamination—and to propose solutions for improving the management of these areas to mitigate the environmental risks associated with military activities (Alasmary, 2025; Barker et al., 2021; Robinson et al., 2008). In this context, and in light of existing knowledge, it appears essential to adopt a similar approach to evaluate the environmental consequences of these training sites, enabling informed actions to mitigate their impact. Indeed, the utilization of lake areas for AAA training in the United States and aviation firing ranges in Switzerland has resulted in the accumulation of significant munition debris and UXO on the lakebeds. These materials, found at different depths, pose multiple risks, including environmental threats to the aquatic ecosystem, potential health impacts from contaminated water and fish consumption, and the danger of UXO explosions (Beck et al., 2018, 2022; Bełdowski et al., 2019; den Otter et al., 2023; Lotufo et al., 2019; Schuster et al., 2021). To address these diverse hazards, authorities have implemented two programs.

The first program focuses on mitigating the risk of UXO explosions. These items, whether underwater or on land, are highly hazardous and can detonate if disturbed, potentially causing harm or damage. To raise public awareness about the potential dangers associated with UXO, both countries have developed prevention campaigns. In the United States, this campaign is known as the “3Rs"—Recognize, Retreat, and Report. It encompasses a wide range of educational resources targeting different audiences and contexts, including construction, maritime activities, and outdoor recreation. Similarly, Switzerland's campaign covers various geographical areas and recreational activities, such as hiking and scuba diving. In both cases, the recommended procedure is to refrain from touching UXO, mark their location, and promptly report them.

The second program focuses on the environmental and health risks associated with underwater munitions, specifically heavy metals like antimony, lead, and copper, as well as explosives such as TNT, RDX, and perchlorates. Conducting environmental assessments is essential to evaluate these risks, and each country has established a legislative framework to govern inland and underwater munition sites. In the United States, the Comprehensive Environmental Response, Compensation, and Liability Act (Public law 96–510), commonly known as the Superfund legislation, mandates assessments of FUDS to determine if further action is needed due to the presence of munitions, explosives, or munitions constituents. In Switzerland, the Ordinance on the Remediation of Polluted Sites (RO 1998 2261), also referred to as the Contaminated Sites Ordinance, ensures remediation if sites pose a threat to human health or the environment.

In Switzerland, initial efforts to assess the potential effects of munitions dumped in Lake Thun date back to the early 1990s (Stucki and Mathieu, 1995). However, the issue resurfaced in 2004, leading to environmental assessments in Lakes Thun, Brienz, and Lucerne (Schenker et al., 2012; Schenker and Werthmüller, 2017, 2020; van Stuijvenberg et al., 2005). More recently, in the summer of 2024, while maintaining the status quo, Switzerland's Federal Office for Defense Procurement (Armasuisse) launched a competition to solicit new ideas for the removal of munitions from the depths of these lakes (Armasuisse, 2024). As for the Forel firing range, its first analysis occurred more than a decade later (Burger et al., 2015), followed by water and sediment analyses six years later (Mathieu et al., 2021). Nevertheless, the Swiss Federal Audit Office determined that these two studies did not meet the criteria for a preliminary technical investigation, as outlined in the Contaminated Sites Ordinance (Eidgenössische Finanzkontrolle, 2022). Consequently, they do not provide sufficient grounds to determine the necessity of monitoring or remediation at the Forel firing range. To comply with current legislation, the Federal Councilor responsible for the DDPS announced an upcoming comprehensive environmental assessment focusing on the impact on flora and fauna, as well as sediment stratification (DDPS, 2021).

In the context of the Great Lakes region, initial research on military waste dumping in Lake Superior dates back to the 1970s (Charrière and Langston, 2023). Concerning Fort Sheridan, it was not until 1996 that the USACE initiated a comprehensive study encompassing munitions and explosives both on land and underwater (USACE, 1996). Subsequently, in response to a 1998 petition advocating for an assessment of the section of Lake Michigan used as an artillery range by the Army as a potential Superfund Site (Pollack, 1998), the U.S. Environmental Protection Agency conducted its initial study of the Fort Sheridan water range in the same year (Muno, 1998). Following these developments, the USACE conducted multiple studies in this area, with the most recent concluding that, despite earlier reassuring findings, only a partial assessment of the water range had taken place, rendering the data non-representative (Engineering-environmental Management, 2005). Consequently, despite extensive studies conducted, significant knowledge gaps remain regarding the environmental impacts of underwater munitions resulting from the historical activities at Fort Sheridan. In contrast to the former Erie Army Depot's water impact area in Lake Erie, which underwent diligent investigations (Mc Donald, 2009), no systematic mapping of the density and general distribution of munition debris and UXO has been conducted in any of the five former AAA water ranges in Lake Michigan.

### Contemporary reflections on military legacy and lakescapes

**Figure 3.**
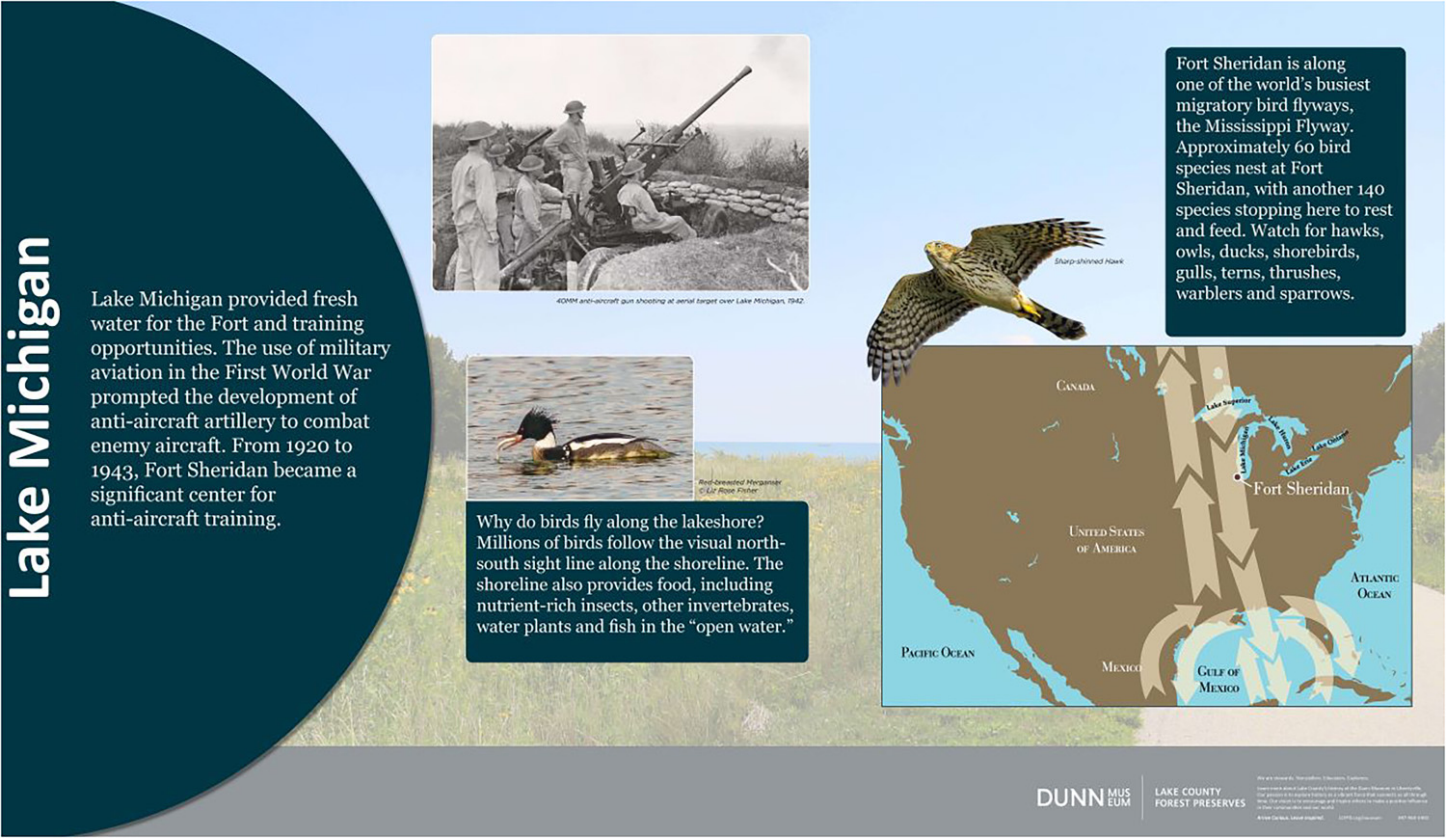
Panels relating to the AAA military training and the lake. Credits: Courtesy of The Lake County Forest Preserves.

In today's context, it is essential to distinguish between the legacies of military activities on land and those on water. Notably, Lake Neuchâtel and Lake Michigan bear historical traces of military operations along their shores. Sites like Camp Logan and Fort Sheridan, both listed in the National Register of Historic Places (National Park Service, 1983, 2000), serve as memorials commemorating the military endeavors conducted there. However, AAA training exercises on Lake Michigan have left no discernible marks, except for one site that commemorates its past training activities. Within the Fort Sheridan Forest Preserve, several outdoor educational exhibits seamlessly integrate with the surrounding landscape. These exhibits recount the military history of Fort Sheridan, with one specifically dedicated to coastal artillery. This exhibit aims to recreate an environment resembling an army training gun emplacement from the 1920s to the 1940s and showcases a replica of an AAA cannon, similar to those once used for training at the Fort. The cannon is strategically positioned along the lakefront. Additionally, an informative board titled “The Lake” features a photograph of a 40 mm anti-aircraft gun shooting in action, firing over Lake Michigan (Figure 3). Accompanying this image is a concise text that underscores Lake Michigan's significance as a vital resource, serving as a source of drinking water and an essential spot on the Mississippi Flyway, a bird migration route.

The situation at Lake Neuchâtel differs significantly. The Forel firing range, which remains operational, has led to the establishment of permanent military facilities such as a boat mooring jetty and a bunker, making the military presence visibly evident on and near the lakeshore. Additionally, temporary military activities, such as aerial shooting, sea survival training, and parachute explorer survival exercises, further underscore the visibility and hazards of military operations. However, the Forel target area and its adjacent shoreline, part of the natural reserve known as the “Grande Cariçaie”, contain numerous rare or endangered plant species. This reserve has been designated under the Ramsar Convention as a wetland of international importance since 1990. To raise public awareness about the fragility of this environment and to preserve it, the DDPS collaborates with the organization managing the “Grande Cariçaie”. This collaboration was formalized through a convention signed on July 20, 2000, and further strengthened with the implementation of the Nature-Landscape-Army program at the Forel firing range (Association de la Grande Cariçaie, 2013). This program aims to regulate military activities to minimize disruptions to natural habitats and wildlife, and to implement measures for biodiversity protection and enhancement. Despite these efforts, munition debris and UXOs continue to accumulate on the lakebed, with no substantial initiatives by the military to adopt environmentally sustainable retrieval and disposal methods. This coexistence of degradation and conservation raises questions and can be viewed as paradoxical or cynical, echoing the concept of “opportunistic conservation” developed by Havlick (2014). According to Havlick,

conservation can thus emerge as a cynical move to cover the tracks of military negligence or as a genuine and creative effort to achieve conservation successes. In either case, we are better served by working to understand these places in their complexity, to engage with them as sites of conservation and militarization, and to do what we can do to ensure that we face the challenges posed by such complex landscapes as comprehensively as possible. (Havlick, 2014: 283)

Another disparity in the approach between military land range management and military water range management must be highlighted. This disparity also applies to UXO management in Canada, where priority is given to land-based sites, even though underwater munitions are not ignored (Souchen, 2023). Despite challenges, former military sites underwent cleanup after their closure to ensure their transition into various development projects (Lubbert and Chu, 2000). However, although a site could include both land and water, aquatic spaces were often overlooked. To illustrate this perspective, consider Camp Haven. After being used by the 5^th^ Army as an AAA firing range from 1949 to 1951, Wisconsin Electric purchased it in 1966 and resold it to another private company, Kohler Inc., in 1995, without any transformation. With the new ownership, a new phase began. Kohler Inc., aiming to convert this site into a golf course and aware of its military history, commissioned an archaeological study to determine if there were any UXOs buried on the site. The study found no trace of UXOs (USACE, 2002b). However, this study solely focused on the terrestrial portion of the site, overlooking the area where the largest quantity of ordnance debris and UXO resides—beneath the lake's surface. A comprehensive investigation encompassing both land and lake areas would have been instrumental in reconstructing the environmental impacts of the training camp as a whole. While the FUDS program tends to address environmental contamination related to past military operations, water range firing assessment is recent and still ongoing at many sites, such as Edithton Beach (USACE, 2017) and Camp Claybanks (Shaw Environmental, 2007).

In Switzerland, this disparity between terrestrial and aquatic sites is also observed during the implementation of the OSites ordinance. Following its implementation, Switzerland has identified 38,000 polluted sites, of which one in ten is a shooting range—military or public—demonstrating that, despite its neutrality, the country bears significant traces of military activity. However, only terrestrial shooting ranges were studied in compliance with the OSites ordinance. Due to the presence of high concentrations of lead and other heavy metals, many of them were declared contaminated, meaning they pose a risk to human health and the environment, and thus had to be remediated (e.g., Guin (canton of Fribourg)) (Fitze and Meuli, 2015). This ordinance was not designed to apply to aquatic environments, making its application to shooting training sites located wholly or partially within a body of surface water challenging for multiple reasons. To address this gap, the Federal Office for the Environment published a guide to provide an overview of the topic as well as practical assistance with enforcing legislation on contaminated sites in conjunction with surface waters (OFEV, 2020). Following the announcement by the Swiss Federal Audit Office highlighting the shortcomings of studies conducted on the Forel firing range in Lake Neuchâtel (Eidgenössische Finanzkontrolle, 2022), the DDPS (2021) decided to deepen these preliminary studies to determine whether the area should be remediated, monitored, or if the ordnance debris and UXOs could continue to rest on the lake bed without risk to the environment.

## Conclusion

This article aims to demonstrate that the relationship between military activities and landscapes can be reexamined through the lens of militarized lakescapes. These sites exemplify hybrid spaces, where military, ecological, and infrastructural dimensions converge. Indeed, the military legacy related to war preparedness extends beyond terrestrial spaces and encompasses aquatic environments. Despite the existence of numerous underwater munition sites worldwide, the study of these aquatic spaces has often been neglected. Recent studies, however, have begun to engage with this topic from diverse and interdisciplinary perspectives. For instance, Neimanis (2020) extends Nixon's (2011) concept of slow violence by applying it to the case of chemical weapons dumped in Sydney's seas after World War II, offering new insights into the environmental afterlives of war. Similarly, Neimanis et al. (2017) underscore the need for sustained interdisciplinary research that addresses both the historical trajectories and the present-day material entanglements of chemical weapons dumped in the Gotland Deep (Baltic Sea) at the end of World War II.

It is essential to consider the geographical characteristics of places bearing the trace of any military activity, whether completed or ongoing, visible or concealed, both inland and over water. For instance, examining the military installation at Gibraltar through the lens of geo-environmental history reveals more than just the visible part of the fortress. Rose (2001) highlights the consequences of military engineers’ construction of specific infrastructures—such as tunnels and caves, which remain hidden from the outside world—on the geology of the Rock of Gibraltar. Similarly, underwater munition sites resulting from military activities, such as training, naval battles, and waste disposal, should be considered as territorial markers or vestiges of past military activities. Each aquatic space and underwater munition site possess distinct characteristics shaped by the nature of military activities, the munitions present, and environmental conditions (Charrière, 2023; Paetzel, 2002).

These sites, due to their unique stratification within lakebeds, function as veritable archaeological sites, offering insights into the evolution of military strategies, technological advancements in military equipment, and changing perceptions and attitudes towards aquatic environments. Therefore, similar to how archaeologists meticulously study remnants of the past to enhance our understanding of previous lifestyles and societal practices, including those related to military activity (Geier et al., 2010; Gustafsson et al., 2017), underwater munition sites represent authentic vestiges of past wars or military preparedness. Collaborating with archaeologists can help address challenges related to data scarcity, as they also grapple with deciphering changes that have occurred at these sites over time and space. The same logic applies to limnologists, who consider sediments as environmental archives. Their research contributes to our understanding of historical aquatic events by providing insights into geological history, climatic fluctuations, environmental changes, and anthropogenic pollution (Gascón Díez et al., 2017; Parrinello and Kondolf, 2021).

Documenting underwater munition sites is crucial not only for historically tracing the entangled relationships between the military world, the civilian world, and the natural environment, but also for addressing contemporary environmental and socio-economic concerns. While scientific knowledge regarding the impacts of such waste on aquatic ecosystems is still evolving (Beck et al., 2018; Cumming 2024; Lotufo et al., 2021), identifying the location and composition of these sites is essential for sustainable environmental management. Indeed, as Souchen notes in the context of underwater munitions at sea and in the ocean,

most experts are in near unanimous agreement that continuous monitoring programs are needed to keep tabs on dumpsites and alert authorities to changing conditions, as scientists cannot preclude the fact that risks to human health and the environment might increase in the future. (2023: 84)

The material presence of ordnance on lakebeds is not inert; it imposes constraints on alternative uses of these spaces—such as cables installations and renewable energy infrastructure like hydrothermal energy grid or even offshore wind farms—thereby highlighting the absence of strict boundaries between military and civilian spaces. These militarized lakescapes, much like their terrestrial counterparts (Pearson, 2012), embody a legacy of war preparedness or conflict that continues to shape socio-ecological conditions and constrain future possibilities. In parallel, the still nascent discipline of underwater or maritime archaeology (Perez-Alvaro, 2019) shows a growing interest in sunken WWII ships—whether lost in combat or deliberately scuttled with conventional and chemical weapons—highlighting the dilemmas of maritime heritage and the environmental risks they pose (Browne, 2019). This emerging body of research reinforces the urgency of addressing aquatic military legacies not only through environmental science and policy but also through cultural and historical lenses.

That said, a global and holistic management approach should be applied to aquatic areas, especially when they are entirely national, rather than a case-by-case management of underwater munition sites, which only leads to a partial understanding of this heritage. To provide a comprehensive assessment of past and present military activities over Lake Michigan, other sites should also be studied. For example, the Naval Station Great Lakes (Illinois), established in 1911 as the Navy's largest training *station*, has conducted firing exercises over Lake Michigan since 1948 (Malcom Pirnie, 2005; Tetra Tech, 2010). Additionally, the “Range 6903” between Port Washington and Manitowoc, Wisconsin, covering an area of 288 square miles of Lake Michigan, has been used for military exercises since the late 1950s, including air-to-air missiles, air-to-air gunnery, rocketry, bombing, and more (USGAO, 1992).

## Highlights

- The environmental consequences of military training activities on inland freshwater ecosystems in the context of war preparedness.
- The transformation of lakescapes by military training and the environmental impacts of such practices on inland freshwater bodies.
- Explanation of the current management—or lack thereof—of the military legacy at the bottom of lakes.

## Notes

1. A complete view of all Formerly Used Defense Sites, including those located around Lake Michigan, can be viewed online at: https://geospatial.sec.usace.army.mil/arcgis/apps/webappviewer/index.html?id=fe3efb63b00e45b5857d3b9e15420f92 (accessed 20 February 2025).

2. The seven categories of “underwater munitions sites” are as follows: (i) ranges and target areas with a variety of munitions; (ii) defense sites including forts; (iii) ocean disposal operations prior to 1972; (iv) accident sites; (v) acts of war such as combat sites or sunken vessels; (vi) former manufacturing sites for military munitions; (vii) handling sites for open burning and open detonation practices (SERDP and ESTCP, 2010).

3. Available online at: https://www.vtg.admin.ch/fr/avis-de-tir-daviation#Fliegerschiessplatz-Neuenburgersee-/-Place-de-tir-d%E2%80%99aviation-du-lac-de-Neuch%C3%A2tel-(LS-R4) (accessed 6 June 2025).

## References

[bibr1-25148486251363734] **Primary sources**

[bibr2-25148486251363734] **Archives**

[bibr8-25148486251363734] Amherd V (2022) Intervention - Tirs militaires aux abords de la réserve naturelle de la Grande Cariçaie. Cela suffit ! Motion 21.3132, National Council, Bern, March 9, 2022. Available at: https://www.parlament.ch/fr/ratsbetrieb/amtliches-bulletin/amtliches-bulletin-die-verhandlungen?SubjectId=56258 (accessed 5 June 2024).

[bibr9-25148486251363734] Armasuisse (2024) Armasuisse launches idea competition for environmentally friendly and safe recovery methods of ammunition from Swiss waters. Press release, Thun, August 7, 2024. Available at: https://www.admin.ch/gov/en/start/documentation/media-releases.msg-id-102016.html (accessed on 20 February 2025).

[bibr38-25148486251363734] Département fédéral de la défense, de la protection de la population et des sports (DDPS) (2021) Place de tir d’aviation de Forel : analyses supplémentaires nécessaires. Press release, Bern, Septembre 29, 2021. Available at: https://www.admin.ch/gov/fr/accueil/documentation/communiques.msg-id-85295.html (accessed 5 June 2024).

[bibr56-25148486251363734] Fridez PA (2021) Tirs militaires aux abords de la réserve naturelle de la Grande Cariçaie. Cela suffit ! Motion 21.3132, National Council, Bern, March 11, 2021. Available at: https://www.parlament.ch/fr/ratsbetrieb/suche-curia-vista/geschaeft?AffairId=20213132 (accessed 5 June 2024).

[bibr66-25148486251363734] Haller U (2004) Repêchage et élimination des munitions déposées au fond des lacs suisses. Motion 04.3220, National Council, Bern, May 5, 2004. Available at: https://www.parlament.ch/en/ratsbetrieb/suche-curia-vista/geschaeft?AffairId=20043220 (accessed 5 June 2024).

[bibr72-25148486251363734] Hurni B (2021) Sites pollués par l’armée. Quelles sont les perspectives d’assainissementPostulate 21.3636, National Council, Bern, June 3, 2021. Available at: https://www.parlament.ch/fr/ratsbetrieb/suche-curia-vista/geschaeft?AffairId=20213636 (accessed 5 June 2024).

[bibr93-25148486251363734] Muno W, Director of Superfund Division, U.S. Environmental Protection Agency (1998) Letter to Steven Pollack. Subject: Petition. December 15, 1998. U.S. Army Corps of Engineers, Omaha District. Steven Pollack personal records, Highland Park (IL).

[bibr112-25148486251363734] Pollack S (1998) Letter to William Muno, Director of Superfund Division, U.S. Environmental Protection Agency. Subject: Petition. June 29, 1998. U.S. Army Corps of Engineers, Omaha District. Steven Pollack personal records, Highland Park (IL).

[bibr3-25148486251363734] **Law**

[bibr51-25148486251363734] Federal Constitution of the Swiss Confederation of May 29, 1874. ETH-Bibliothek Zürich. Rar 6562.

[bibr52-25148486251363734] Federal Constitution of the Swiss Confederation of September 12, 1848. ETH-Bibliothek Zürich. Rar 38263.

[bibr102-25148486251363734] Ordinance on the Remediation of Polluted Sites (Contaminated Sites Ordinance, OSites) of 26 August 1998. RO 1998 2261. RS 814.680.

[bibr115-25148486251363734] Public land order 237 – Michigan: Withdrawal of public lands for use of Navy Department (9 FR 7526). Federal Register, the National Archives of the United States (1944). Volume 9, Number 134, p. 7526.

[bibr114-25148486251363734] Public land order 1279 – Michigan: Revoking Public Land Order No. 237 of June 22, 1944 (21 FR 2244). Federal Register, the National Archives of the United States (1956). Volume 21, Number 67, p. 2244.

[bibr116-25148486251363734] Public law 96-510 – Comprehensive Environmental Response, Compensation, and Liability Act of 1980 (H.R. 7020). 94 Stat. 2767.

[bibr117-25148486251363734] Regulation No. 200-3-1 – Environmental Quality. Formerly Used Defense Sites (FUDS) Program Policy. Department of the Army, U.S. Army Corps of Engineers. Washignton, May 10, 2004.

[bibr4-25148486251363734] **Newspapers/Magazine**

[bibr25-25148486251363734] Chicago Daily Tribune (1931) Fort Sheridan anti-aircraft guns to blaze at targets. 12 June, 4.

[bibr26-25148486251363734] Chicago Daily Tribune (1940a) Anti-Aircraft Training Opens at Fort Sheridan – 500 Cadets, Reservists Get 6 Weeks Course. 18 June, 15.

[bibr27-25148486251363734] Chicago Daily Tribune (1940b) The Graphic laboratory of Popular Science – The Story of Anti-Aircraft Weapons. 7 July, 6.

[bibr28-25148486251363734] Chicago Daily Tribune (1949) Illinois AAA brigade moves to camp haven – Couldn’t fire guns at McCoy. 24 August, 21.

[bibr29-25148486251363734] Chicago Daily Tribune (1953) Army defends Michigan site for AAA range. 17 June, 19.

[bibr30-25148486251363734] Chicago Daily Tribune (1961) Danger area. 13 April, 8.

[bibr39-25148486251363734] Dippel B (2017) Before whistling straits, there was camp haven. The Sheboygan Press. Available at: https://www.sheboyganpress.com/story/life/2017/07/28/before-whistling-straits-there-camp-haven/521018001/ (accessed 13 June 2024).

[bibr45-25148486251363734] Duluth News-Tribune (1945) Cartridge dumping is safety measure. October 3, 5.

[bibr53-25148486251363734] Fitze U Meuli K (2015) Petites sources, grandes pollutions. *Les ressources naturelles en Suisse*. Environnement 4: 23–25.

[bibr59-25148486251363734] Gazette de Lausanne (1929) Chronique Militaire - L’aviation militaire à Payerne. 22 June, 4.

[bibr60-25148486251363734] Gazette de Lausanne (1954) L’empereur d’Ethiopie reçu à Genève. 29 November, 1, 8.

[bibr74-25148486251363734] Hutchings H (1940) Fort Sheridan rehearses for military show – Public to view guns and see them in action. Chicago Daily Tribune, 22 September, 13.

[bibr98-25148486251363734] New York Times (1945) British sink poison gas in Sea West of Ireland. 7 September, 3.

[bibr142-25148486251363734] The Montague Observer (1953) New anti-aircraft gun range named camp Claybanks. 23 April, 1.

[bibr143-25148486251363734] The Sheboygan Press (1956) Exclude Harbor Entrance from Danger Area Resulting from Firing at Camp Haven. 10 April, 6.

[bibr144-25148486251363734] The True Republicain (1931) Coast artillery open aerial target practices. 7 October, 2.

[bibr145-25148486251363734] Thomis W (1939) Thousands see nation’s aerial peace defense. Chicago Daily Tribune, 11 October, 1, 8.

[bibr146-25148486251363734] Thompson JH (1955) Lake Michigan mist hinders guard gunners - writer finds Whitehall camp ‘primitive’. Chicago Daily Tribune, 16 July, 14.

[bibr148-25148486251363734] Turner FH (1953) Voice of the people - The army takes over a resort. Chicago Daily Tribune, 4 July, 9.

[bibr155-25148486251363734] Vipond J (1945) Dumped high explosives in Georgian Bay: Ancient ammo racked in old Davey’s locker. Globe and Mail, 21 November, 15.

[bibr5-25148486251363734] **Reports**

[bibr10-25148486251363734] Armée Suisse (2021) *100 ans. École de recrues base aérienne, 1921-*2021. Forces aériennes, Payerne, Switzerland.

[bibr12-25148486251363734] Association de la Grande Cariçaie (2013) NPA – Place de tirs de Forel. Programme de mise en œuvre des mesures Nature et Paysage 2012-2023. Final version, May 28. Cheseaux-Noréaz, Switzerland.

[bibr22-25148486251363734] Burger M Jakob A Stauffer M (2015) Analysebericht UA2015-16. Report for the Federal Office for Civil Protection – Spiez Laboratory, Bern – Spiez, Switzerland.

[bibr35-25148486251363734] Defense Secretary’s Commission (1988) Base Realignments and Closures. Washington, D.C.

[bibr37-25148486251363734] Département fédéral de la défense, de la protection de la population et des sports (DDPS) (2004) Étude historique concernant le dépôt et l’immersion de munitions dans les lacs suisses – Résumé. Bern, Switzerland.

[bibr49-25148486251363734] Eidgenössische Finanzkontrolle (2022) Prüfung des Umgangs mit Altlasten. Eidgenössisches Departement für Verteidigung, Bevölkerungsschutz und Sport. EFK-21545. Bern, Switzerland.

[bibr50-25148486251363734] Engineering-environmental Management (2005) Stakeholder Draft of the Military Munitions Response Program Historical Records Review Fort Sheridan, Illinois. Report for the U.S. Army Corps of Engineers, Omaha District. Englewood, CO.

[bibr55-25148486251363734] Forces aériennes (2009) Place de tirs de Forel. Projet Nature-Paysage-Armée. Report for the Association de la Grande Cariçaie. Office fédéral des exploitations, Payerne, Switzerland.

[bibr57-25148486251363734] Gächter D Cervera G Dériaz C (2004) Investigations historiques relatives aux dépôts et aux immersions de munitions dans les lacs suisses – Lot 1 : Suisse Romande. Report for the Swiss Federal Department of Defense, Civil Protection, and Sport. Bern, Switzerland.

[bibr62-25148486251363734] GEO Consultants (2009) Preliminary Assessment Report for Formerly Camp Logan Military Reservation, Zion, Illinois. Report for the U.S. Army Corps of Engineers, Louisville District. Contract No. W912QR-04-D0030, February. Kevil, KY.

[bibr75-25148486251363734] HydroGeoLogic (2013) Final Preliminary Assessment/Site Inspection Former Waugoshance Point Target, Emmet County, Michigan. Report for the U.S. Army Corps of Engineers, Louisville District. Contract No. W9128F-10-0057, June. Ballston Lake, NY.

[bibr81-25148486251363734] Lotufo GR Rosen G Carton G (2021) SERDP and ESTCP Workshop on State of the Science and Research and Development Needs for Assessing the Environmental Risks of Conventional Underwater Military Munitions. SERDP Project ER-2341. U.S. Army Engineer Research and Development Center, Vicksburg, MS.

[bibr86-25148486251363734] Malcom Pirnie (2005) Final Water Area Munitions Study. Naval Station Great Lakes, Illinois. Naval Training Center Lakefront. Report for the Naval Station Great Lakes Commander, Naval Training Center. Contract No. N62472-02-D-1300, April. Columbus, OH.

[bibr89-25148486251363734] Mathieu M Pasche S Stauffer M , et al. (2021) Analyses de l’eau et des sédiments effectuées en mars 2021 sur la place de tir des Forces aériennes de Forel. Report for the General Secretariat of the Swiss Federal Department of Defense, Civil Protection, and Sport. Thun, Switzerland.

[bibr90-25148486251363734] Mc Donald JR (2009) *The MTA UXO Survey and Target Recovery on Lake Erie at the Former Erie Army Depot*. Science Applications International Corporation, Cary, NC.

[bibr94-25148486251363734] National Park Service (1983) Fort Sheridan Historic District, Lake County, Illinois. National Register of Historic Places Inventory–Nomination Form. U.S. Department of the Interior, Washington, D.C.

[bibr95-25148486251363734] National Park Service (2000) Camp Logan National Guard Rifle Range Historic District, Lake County, Illinois. National Register of Historic Places Inventory–Nomination Form. U.S. Department of the Interior, Washington, D.C.

[bibr101-25148486251363734] Office fédéral de l’environnement (OFEV) (2020) Indemnisations en vertu de l’OTAS pour les installations de tir. Communication de l’OFEV en tant qu’autorité d’exécution. 4th edition. L’environnement pratique n°063. Bern, Switzerland.

[bibr110-25148486251363734] Philipp R (2004) Historische Abklärungen zu Ablagerungen und Munitionsversenkungen in Schweizer Seen; Qualitätssicherung,Vorlagen. Rapport magma SA 04 103.4. Report for the General Secretariat of the Swiss Federal Department of Defense, Civil Protection, and Sport. Bern, Switzerland.

[bibr125-25148486251363734] Schenker F Lancini A van Stuijvenberg J (2012) Militärische Munitionsversenkungen in Schweizer Seen – Umfassende Gefährdungsabschätzung ergänzt mit Abklärungen zur Herkunft von Spurenbelastungen durch Explosivstoffe. Report for the Swiss Federal Department of Defense, Civil Protection, and Sport and the environmental agencies of the cantons of Bern, Lucerne, Nidwalden, Schwyz, and Uri. Bern, Switzerland.

[bibr126-25148486251363734] Schenker F Werthmüller S (2017) Militärische Munitionsversenkungen in Schweizer Seen – Explosivstoffmonitoring 2012-2016. Report for the Swiss Federal Department of Defense, Civil Protection, and Sport and the environmental agencies of the cantons of Bern, Lucerne, Nidwalden, Schwyz, and Uri. Bern, Switzerland.

[bibr127-25148486251363734] Schenker F Werthmüller S (2020) Militärische Munitionsversenkungen in Schweizer Seen – Bericht zum Explosivstoffmonitoring 2019 mit Vergleich zu den Messungen 2009. Report for the Swiss Federal Department of Defense, Civil Protection, and Sport and the environmental agencies of the cantons of Bern, Lucerne, Nidwalden, Schwyz, and Uri. Bern, Switzerland.

[bibr129-25148486251363734] Shaw Environmental (2007) Final Site Inspection Report. Camp Claybanks AAA Firing Range, FUDS Property No. E05MI0034, Oceana County, Michigan. Report for the U.S Department of the Army and U.S. Army Corps of Engineers, Omaha District. Contract No. W912DY-04-D-0010, September. Denver, CO.

[bibr130-25148486251363734] Shaw Environmental (2009) Final Site-Specific Inspection Report. Edithon Beach Anti-Aircraft Artillery Training Center Kenosha County, WI. Report for the U.S Department of the Army and U.S. Army Corps of Engineers, Omaha District. Contract No. W912DY-04-D-0010, September. Denver, CO.

[bibr131-25148486251363734] Shaw Environmental (2011) Final Site-Specific Inspection Report. Hat Island Target, FUDS Property No. E05MI1208, Charlevoix County, Michigan. Report for the U.S Department of the Army and U.S. Army Corps of Engineers, Omaha District. Contract No. W912DY-04-D-0010, October. Denver, CO.

[bibr137-25148486251363734] Strategic Environmental Research and Development Program (SERDP) and Environmental Security Technology Certification Program (ESTCP) (2010) Munitions in the Underwater Environment: State of the Science and Knowledge Gaps. White Paper. Arlington, VA.

[bibr138-25148486251363734] Stucki H Mathieu J (1995) Schlussbericht zur Untersuchung der Munitionsdeponien im Thunersee. Report for the Swiss Federal Department of Defense, Civil Protection, and Sport. Thun, Switzerland.

[bibr139-25148486251363734] Suter H (2004) Fliegermunition in CH Seen durch Schiessbetrieb, Stand: Ende 2003. Prepared for GADZ Géotechnique Appliquée Dériaz SA. Forces aériennes, Payerne, Switzerland.

[bibr141-25148486251363734] Tetra Tech (2010) Site Inspection Report. Munitions Response Program Ranges. Naval Station Great Lakes, Illinois. Comprehensive Long-Term Environmental Action Navy (Clean) Contract. Report for the Naval Facilities Engineering Command Midwest. Contract No. N62472-03-D-0057, September. King of Prussia, PA.

[bibr147-25148486251363734] Thomson HC Mayo L (1991) United States Army in World War II. The Technical Services. The Ordnance Department: Procurement and Supply. Center of Military History, United States Army, Washington, D.C.

[bibr150-25148486251363734] U.S. Army Corps of Engineers (USACE), St. Louis District (1996) Archives Search Report Findings, Fort Sheridan, Lake County, Illinois. U.S. Department of Defense Program Base Realignment and Closure Ordnance, Ammunition and Explosives. St Louis, MO.

[bibr151-25148486251363734] U.S. Army Corps of Engineers (USACE), St. Louis District (2002a) *Defense Environmental Restoration Program for Formerly Used Defense Sites. Ordnance and Explosives. Archives Search Report. Findings. Edithton Beach Anti-Aircraft Artillery Training Center, Kenosha County, Wisconsin, Project N° E05WI088301*. Final. Report for the U.S. Army Corps of Engineers, Huntsville Engineering and Support Center. St Louis, MO.

[bibr152-25148486251363734] U.S. Army Corps of Engineers (USACE), St. Louis District (2002b) *Defense Environmental Restoration Program for Formerly Used Defense Sites. Ordnance and Explosives. Archives Search Report. Camp Havens Anti-Aircraft Artillery Firing Range, Sheboygan County, Wisconsin. Project Number – E05WI506903*. Final. Report for the U.S. Army Corps of Engineers, Huntsville Engineering and Support Center. St Louis, MO.

[bibr149-25148486251363734] U.S. Army Corps of Engineers (USACE), Omaha District (2017) *Formerly Used Defense Sites Interim Risk Management Communication Assessment Summary. Edit Bea AA Trg Cen AAA Range - Water. FUDS Project No. E05WI088301. Kenosha County, Wisconsin*. Omaha, NE.

[bibr153-25148486251363734] U.S. General Accounting Office (USGAO) (1992) Military Training. Unexploded Ordnance Found in Lake Michigan. Report to the Honorable F. James Sensenbrenner, Jr., House of Representatives. GAO/NSIAD-92-95. Washington, D.C.

[bibr154-25148486251363734] van Stuijvenberg J Schenker F Lancini A (2005) *Gefährdungsabschätzung zu militärischen Munitionsversenkungen in Schweizer Seen; Zusammenstellung aller verfügbaren Daten bezüglich Brienzer-, Thuner- und Urnersee, sowie für das Gersauerbecken des Vierwaldstättersees*. Report for the Swiss Federal Department of Defense, Civil Protection, and Sport. Thun, Switzerland.

[bibr6-25148486251363734] **Secondary sources**

[bibr7-25148486251363734] Alasmary Z (2025) Lead (pb) contamination in soil and plants at military shooting ranges and its mitigation strategies: A comprehensive review. Processes 13(2): 345.

[bibr11-25148486251363734] Ashworth W (1987) The Late, Great Lakes. An Environmental History. Detroit, MI: Wayne State University Press.

[bibr13-25148486251363734] Bardati DR , 1996. The view from shore: The impact of boating on lakescape aesthetics . Master of Arts Thesis, McGill University, Canada.

[bibr14-25148486251363734] Barker AJ Clausen JL Douglas TA , et al. (2021) Environmental impact of metals resulting from military training activities: A review. Chemosphere 265: 129110. 33272677 10.1016/j.chemosphere.2020.129110

[bibr16-25148486251363734] Beck AJ Gledhill M Schlosser C , et al. (2018) Spread, behavior, and ecosystem consequences of conventional munitions compounds in coastal marine waters. Frontiers in Marine Science 5: 141.

[bibr15-25148486251363734] Beck AJ Gledhill M Kampmeier M , et al. (2022) Explosives compounds from sea-dumped relic munitions accumulate in marine biota. Science of the Total Environment 806(4): 151266. 34757098 10.1016/j.scitotenv.2021.151266

[bibr17-25148486251363734] Bełdowski J Szubska M Siedlewicz G , et al. (2019) Sea-dumped ammunition as a possible source of mercury to the baltic sea sediments. Science of the Total Environment 674: 363–373. 31005838 10.1016/j.scitotenv.2019.04.058

[bibr18-25148486251363734] Biggs D (2018) Footprints of War. Militarized Landscapes in Vietnam. Seattle, WA: University of Washington Press.

[bibr19-25148486251363734] Bouleau G (2014) The co-production of science and waterscapes: The case of the seine and the rhône rivers, France. Geoforum; Journal of Physical, Human, and Regional Geosciences 57: 248–257.

[bibr20-25148486251363734] Brewer P Nakayama N (2008) What lies beneath: A plea for complete information. Environmental Science and Technology 42(5): 1394–1399. 18441779 10.1021/es087088h

[bibr21-25148486251363734] Browne K (2019) “Ghost battleships” of the pacific: Metal pirates, WWII heritage, and environmental protection. Journal of Maritime Archaeology 14(1): 1–28.

[bibr23-25148486251363734] Charrière E (2023) Les immersions de munitions dans les lacs suisses et français (1919-aujourd’hui), de l’oubli à un statu quo évolutif. Bruxelles: Peter Lang.

[bibr24-25148486251363734] Charrière E Langston N (2023) Dumping military waste into lake superior: The historic legacies of secrecy, censorship, and uncertainty. Water History 15: 173–200. 37649726 10.1007/s12685-023-00329-y PMC10462539

[bibr31-25148486251363734] Cornea N Zimmer A Véron R (2016) Ponds, power and institutions: The everyday governance of accessing urban water bodies in a small bengali city. International Journal of Urban and Regional Research 40(2): 395–409.

[bibr32-25148486251363734] Cumming A (2024) Munitions underwater – a problem for today. Propellants, Explosives, Pyrotechnics 49(4): e202400052.

[bibr33-25148486251363734] Davis JS (2007) Military natures: Militarism and the environment. GeoJournal 69(3): 131–134.

[bibr34-25148486251363734] Davis JS Hayer-Conroy JS Jones JM (2007) Military pollution and natural purity: Seeing nature and knowing contamination in Vieques, Puerto Rico. GeoJournal 69: 165–179.

[bibr36-25148486251363734] den Otter JH Pröfrock D Bünning TH , et al. (2023) Release of ammunition-related compounds from a Dutch marine dump site. Toxics 11(3): 238. 36977003 10.3390/toxics11030238 PMC10055382

[bibr40-25148486251363734] Doe III WW (2011a) The legacy of federal military land in the US. A geographic retrospective. In: Galgano F Palka EJ (eds) Modern Military Geography. New York, NY: Routledge, 92–103.

[bibr41-25148486251363734] Doe III WW (2011b) Military land as spatial analogs of a twenty-first-century army. Natural environments for testing and training. In: Galgano F Palka EJ (eds) Modern Military Geography. New York, NY: Routledge, 264–277.

[bibr42-25148486251363734] Dreiseitl H Grau D (eds) (2009) New Waterscapes: Planning, Building and Designing with Water. Berlin and Boston, MA: Birkhäuser.

[bibr43-25148486251363734] Dretske D (2007) Lake County, Illinois. An Illustrated History. Lake County, IL: Lake County Discovery Museum.

[bibr44-25148486251363734] Dudley M (2013) Traces of conflict: Environment and eviction in British military training areas, 1943 to present. Journal of War & Culture Studies 6(2): 112–126.

[bibr46-25148486251363734] Dycus S (1996) National Defense and the Environment. Hanover, NH: University Press of New England.

[bibr47-25148486251363734] Edgington RH (2014) Range Wars: The Environmental Contest for White Sands Missile Range. Lincoln, NE: University of Nebraska Press.

[bibr48-25148486251363734] Egloff M Kern G (2010) Lac de Neuchâtel. In Dictionnaire historique de la Suisse. Available at: https://hls-dhs-dss.ch/fr/articles/008667/2010-11-09/ (accessed 4 August 2024).

[bibr54-25148486251363734] Flaminio S Rouillé-Kielo G Le Visage S (2022) Waterscapes and hydrosocial territories: Thinking space in political ecologies of water. Progress in Environmental Geography 1(1-4): 33–57.

[bibr58-25148486251363734] Gascón Díez E Corella JP Adatte T , et al. (2017) High-resolution reconstruction of the 20th century history of trace metals, major elements, and organic matter in sediments in a contaminated area of Lake Geneva, Switzerland. Applied Geochemistry 78: 1–11.

[bibr61-25148486251363734] Geier CR Babits LE Scott DD , et al. (eds) (2010) The Historical Archaeology of Military Sites: Method and Topic. College Station, TX: Texas A&M University Press.

[bibr63-25148486251363734] Gibbes C Havlick DG Robb JR (2017) Land use and land cover in a transitioning militarized landscape. Journal of Land use Science 12(2-3): 182–196.

[bibr64-25148486251363734] Grusky S (1991) The U.S. Navy and Vieques, Puerto Rico: Conflict and coexistence. Canadian Journal of Latin American and Caribbean Studies / Revue Canadienne Des Études Latino-Américaines et Caraïbes 16(31): 105–122.

[bibr65-25148486251363734] Gustafsson A Iglesias Camargo J Karlsson H , et al. (2017) Material histories of the Missile Crisis (1962): Cuban examples of a Soviet nuclear missile hangar and US Marston Mats. Journal of Contemporary Archaeology 4(1): 39–58.

[bibr67-25148486251363734] Havlick DG (2014) Opportunistic conservation at former military sites in the United States. Progress in Physical Geography: Earth and Environment 38(3): 271–285.

[bibr68-25148486251363734] Havlick DG (2018) Bombs Away. Militarization, Conservation, and Ecological Restoration. Chicago, IL: The University of Chicago Press.

[bibr69-25148486251363734] Heinz TL (2005) From civil rights to environmental rights: Constructions of race, community, and identity in three African American newspapers’ coverage of the environmental justice movement. Journal of Communication Inquiry 29(1): 47–64.

[bibr70-25148486251363734] Herzog TR (1985) A cognitive analysis of preference for waterscapes. Journal of Environmental Psychology 5: 225–241.

[bibr73-25148486251363734] Hurst E Ellis R Karippal AB (2022) Lively water infrastructure: Constructed wetlands in more-than-human waterscapes. Environment and Planning E: Nature and Space 8(1): 77–99.

[bibr71-25148486251363734] Hundley N (1987) California’s original waterscape: Harmony & manipulation. California History 66(1): 2–11.

[bibr76-25148486251363734] Jenks A (2007) Model city USA: The environmental costs of victory in World War II and the Cold War. Environmental History 12(3): 552–577.

[bibr77-25148486251363734] Karpouzoglou T Vij S (2017) Waterscape: A perspective for understanding the contested geography of water. WIRES Water 4: e1210.

[bibr79-25148486251363734] Langston N (2021) Climate Ghosts: Migratory Species in the Anthropocene. Waltham, MA: Brandeis University Press.

[bibr78-25148486251363734] Lahtinen R Vuorisalo T (2004) It’s war and everyone can do as they please!’: An environmental history of a Finnish city in wartime. Environmental History 9(4): 679–700.

[bibr80-25148486251363734] Lotufo GR George RD Belden JB , et al. (2019) Release of munitions constituents in aquatic environments under realistic scenarios and validation of polar organic chemical integrative samplers (POCIS) for monitoring. Environmental Toxicology and Chemistry 38: 2383–2391. 31365142 10.1002/etc.4553

[bibr82-25148486251363734] Lubbert RF Chu TJ (2000) Challenges to cleaning up formerly used defense sites in the twenty-first century. Federal Facilities Environmental Journal 11: 5–18.

[bibr83-25148486251363734] Lunstrum L Brady L (2025) Intro Theme issue on Militarized Landscapes. Environment and Planning E: Nature and Space. In review.

[bibr84-25148486251363734] MacFarlane D (2024) The lives of Lake Ontario. An Environmental History. Montréal: McGill-Queen’s University Press.

[bibr85-25148486251363734] Machlis GE Hanson T (2008) Warfare ecology. BioScience 58(8): 729–736.

[bibr87-25148486251363734] Márquez L Fernández Porto J (2000) Vieques: Environmental and ecological damage. Diálogo 4(1): article 7.

[bibr88-25148486251363734] Martini EA (ed) (2015) Proving Grounds. Militarized Landscapes, Weapons Testing, and the Environmental Impact of U.S. Bases. Seattle, WA: University of Washington Press.

[bibr91-25148486251363734] McNeill JR (2021) Les destructions de l’environnement. In: Cabanes B (dir) Dans une histoire de la guerre. Du XIX^e^ siècle à nos jours. Paris: Éditions du Seuil, 99–112.

[bibr92-25148486251363734] Minler N Lane P Taylor B , et al. (2011) Star carr in a postglacial lakescape: 60 years of research. Journal of Wetland Archaeology 11: 1–19.

[bibr96-25148486251363734] Neimanis A (2020) The chemists’ war’ in Sydney’s seas: Water, time, and everyday militarisms. Environment and Planning E: Nature and Space 4(2): 337–353.

[bibr97-25148486251363734] Neimanis A Neimanis A Åsberg C (2017) Fathoming chemical weapons in the Gotland Deep. Cultural Geographies 24(4): 631–638.

[bibr99-25148486251363734] Neyland RS (2002) Preserving and interpreting the archaeology of the United States navy. In: Ruppé CV Barstad JF (eds) International Handbook of Underwater Archaeology. Boston, MA: Springer, 765–781.

[bibr100-25148486251363734] Nixon R (2011) Slow Violence and the Environmentalism of the Poor. Cambridge, MA: Harward University Press.

[bibr104-25148486251363734] O’Sullivan AM Devito KJ D’Orangeville L , et al. (2021) The waterscape continuum concept: Rethinking boundaries in ecosystems. WIRES Water 9: e1598.

[bibr103-25148486251363734] Orlove B Caton SC (2010) Water sustainability: Anthropological approaches and prospects. Annual Review of Anthropology 39: 401–415.

[bibr105-25148486251363734] Paetzel M (2002) Deep marine munition dump sites: Example from arendal, Norway. In: Missiaen T Henriet JP (eds) Chemical Munition Dump Sites in Coastal Environments. Brussels/Ghent: Renard Centre of Marine Geology/University of Ghent, 133–144.

[bibr106-25148486251363734] Parrinello G Kondolf GM (2021) The social life of sediment. Water History 13: 1–12.

[bibr107-25148486251363734] Pearson C (2012) Researching militarized landscapes: A literature review on war and the militarization of the environment. Landscape Research 37(1): 115–133.

[bibr108-25148486251363734] Pearson C Coates P Cole T (eds) (2010) Militarized Landscapes. From Gettysburg to Salisbury Plain. London: Continuum.

[bibr109-25148486251363734] Perez-Alvaro E (2019) Underwater Cultural Heritage: Ethical Concepts and Practical Challenges. London: Routledge.

[bibr111-25148486251363734] Pike DL (2017) Cold war reduction: The principle of the Swiss bunker fantasy. Space and Culture 20(1): 94–106.

[bibr113-25148486251363734] Potocka I (2013) The lakescape in the eyes of a tourist. Quaestiones Geographicae 32(3): 85–97.

[bibr118-25148486251363734] Reno J (2019) Military Waste: The Unexpected Consequence of Permanent War Readiness. Oakland, CA: University of California Press.

[bibr119-25148486251363734] Robinson BH Bischofberger S Stoll A , et al. (2008) Plant uptake of trace elements on a Swiss military shooting range: Uptake pathways and land management implications. Environmental Pollution 153(3): 668–676. 17949872 10.1016/j.envpol.2007.08.034

[bibr120-25148486251363734] Rose EPF (2001) Military engineering on the Rock of Gibraltar and its geoenvironmental legacy. In: Ehlen J Harmon RS (eds) The Environmental Legacy of Military Operations. Boulder, CO: The Geological Society of America, 95–121.

[bibr121-25148486251363734] Ross FE , 2012. The Archaeology of Swiss Neutrality: The defences of the ‘Toblerone Trail’ . PhD Thesis, University of Bristol, United Kingdom.

[bibr122-25148486251363734] Russell E (2010) Afterword: Militarized landscapes. In: Pearson C Coates P Cole T (eds) Militarized Landscapes. From Gettysburg to Salisbury Plain. London: Continuum, 229–237.

[bibr123-25148486251363734] Sanderson H Fauser P Stauber RS , et al. (2017) Civilian exposure to munitions-specific carcinogens and resulting cancer risks for civilians on the Puerto Rican island of vieques following military exercises from 1947 to 1998. Global Security 2(1): 40–61.

[bibr124-25148486251363734] Šantrůčková M Salašová A Sokolová K , et al. (2020) Mapping military landscape as a cultural heritage: Case studyof the Austerlitz/Slavkov battlefield site. AUC Geographica 55(1): 66–76.

[bibr128-25148486251363734] Schuster R Strehse JS Ahvo A , et al. (2021) Exposure to dissolved TNT causes multilevel biological effects in Baltic mussels (Mytilus spp.). Marine Environmental Research 167: 105264. 33725510 10.1016/j.marenvres.2021.105264

[bibr132-25148486251363734] Sheboygan County Historical Research Center (2019) Camp Haven. Sheboygan County’s Anti-aircraft training base. Coppell, TX: Independently Published.

[bibr133-25148486251363734] Sicard JH (2018) Shelling lake Michigan. The short life of camp claybanks. Michigan History 6: 18–21.

[bibr134-25148486251363734] Souchen A (2021) Missing from the record: Historians, archival research and underwater munitions. Rethinking History 25(3): 347–371.

[bibr135-25148486251363734] Souchen A (2023) Out with the old: Munitions disposal, marine environments, and the Canadian military. Scientia Canadensis 45(1): 68–90.

[bibr136-25148486251363734] Stanford JA LC A Whited DC (2017) Riverscapes. In: Hauer FR Lamberti GA (eds) Methods in Stream Ecology, Volume 1. Ecosystem Structure. Boston, MA: Academic Press, 3–19.

[bibr140-25148486251363734] Swyngedouw E (1999) Modernity and hybridity: Nature, *Regeneracionismo*, and the production of the spanish waterscape, 1890-1930. Annals of the Association of American Geographers 89(3): 443–465.

[bibr156-25148486251363734] Wise JE Jr (1989) The Sinking of the UC-97. Naval History Magazine 3(1): 13–14.

[bibr157-25148486251363734] Woodward R (2004) Military Geographies. Oxford: Blackwell Publishing.

[bibr158-25148486251363734] Woodward R (2010) Military Landscapes / Militære Landskap: The military landscape photography of Ingrid Book and Carina Hedén. In: Pearson C Coates P Cole T (eds) Militarized Landscapes: Comparative Histories and Geographies. London: Continuum, 21–38.

[bibr159-25148486251363734] Zentelis R Banks S Roberts JD , et al. (2017) Managing military training-related environmental disturbance. Journal of Environmental Management 204: 486–493. 28930693 10.1016/j.jenvman.2017.09.029

